# Strengthening patient safety in transitions of care: an emerging role for local medical centres in Norway

**DOI:** 10.1186/s12913-016-1708-8

**Published:** 2016-08-30

**Authors:** Trond Kongsvik, Kristin Halvorsen, Tonje Osmundsen, Gudveig Gjøsund

**Affiliations:** 1 NTNU Social Research, Dragvoll Allé 38b, N-7491 Trondheim, Norway; 2 Department of Industrial Economics and Technology Management, Norwegian University of Science and Technology, NTNU, N-7491 Trondheim, Norway

**Keywords:** Patient safety, Competence, Quality, Local medical centres

## Abstract

**Background:**

Patient safety has gained less attention in primary care in comparison to specialised care. We explore how local medical centres (LMCs) can play a role in strengthening patient safety, both locally and in transitions between care levels. LMCs represent a form of intermediate care organisation in Norway that is increasingly used as a strategy for integrated care policies. The analysis is based on institutional theory and general safety theories.

**Methods:**

A qualitative design was applied, involving 20 interviews of nursing home managers, managers at local medical centres and administrative personnel.

**Results:**

The LMCs mediate important information between care levels, partly by means of workarounds, but also as a result of having access to the different information and communications technology (ICT) systems in use. Their knowledge of local conditions is found to be a key asset. LMCs are providers of competence and training for the local level, as well as serving as quality assurers.

**Conclusions:**

As a growing organisational form in Norway, LMCs have to legitimise their role in the health care system. They represent an asset to the local level in terms of information, competence and quality assurance. As they have overlapping competencies, tasks and responsibilities with other parts of the health care system, they add to organisational redundancy and strengthen patient safety.

## Background

Receiving health care services is not without risk. Related to specialised health care, an estimated 8–12 % of patients admitted to hospitals in the European Union (EU) suffer from adverse events while receiving health care [1]. In Norwegian hospitals, it was found that 13 % of in-house patients were injured in 2013 as a consequence of the treatment received [2]. Patient safety can be defined as “…the reduction of risk of unnecessary harm associated with healthcare to an acceptable minimum” [3]. The research on patient safety in *primary* health care is scarce and not consistent. Still, based on a review of available studies, the Health Foundation [4] estimates an adverse event rate of 1–2 % and a prescription error rate around 11 %.

Partly as a response to such numbers, there have been several initiatives to strengthen patient safety and quality in health care during the last decade, as well as on the transnational level, such as through the World Health Organization’s (WHO) Patient Safety Program and the Patient Safety and Quality of Care Working group of the EU. In Norway, there have been extensive efforts the last 5 years to work systematically with patient safety, not least with the national campaign *In Safe Hands 24–7* that has evolved into a long-term government programme [5]. The effort, commissioned by the Ministry of Health, aims to reduce patient harm, build sustainable structures for patient safety, and improve patient safety culture.

Patient safety is also a key focus of the larger healthcare reforms we find under the heading “Integrated Care”. As a political and organisational strategy, integrated care has been a widely used prescription for improving the health care systems in a number of countries [6]. In Norway, integrated care has been formalised on a governmental level through the Coordination Reform, officially launched in 2012. The reform aims to improve quality of care, promote collaboration between health care levels, and provide patient-centred services closer to the patient’s home [7, 8].

Still, patient safety initiatives, as well as patient safety research, have tended to target specialist health care [9, 10], with focus areas such as safe surgery, medication reconciliation, and prevention of infections and pressure ulcers. Primary health care, taking place in diverse settings across 428 municipalities in Norway, has not been involved to the same extent in the patient safety strategies. This implies that, although there might be high-quality patient safety work taking place in individual institutions or communities, there is a gap between primary care and specialist care in terms of systematic patient safety work.

The establishment of Local Medical Centres (LMCs), as intermediate units between primary and specialist health services, is one of the strategies for offering specialist care outside of regional hospitals and for providing greater continuity of care for the patient. When the patient receives care closer to home, strenuous and potentially harmful transportation can be avoided. Carers have easier access to visiting and supporting patients, and specialist care can be given in closer contact with other care services at the local level. LMCs provide healthcare services in the pathway from hospital to home, before, after, or instead of treatment at the hospital [11]. LMCs can be seen as a means for carrying out important aspects of the Coordination Reform, bridging primary and specialist health care delivery by bringing specialised services closer to the municipalities [12].

In this article we will explore how the LMCs as decentralised intermediate care units in Norway, might contribute to strengthening patient safety in primary care. Our research question is: “How may LMCs play a role in strengthening patient safety at the local primary care level, as well as in transition between care levels?” Based on interviews with managers of LMCs and with municipal health care providers in one of the four administrative health regions in Norway, we discuss the potential of LMCs for improving patient safety in primary care. We find that LMCs engage in three main areas related to patient safety, namely *information exchange*, *competence building*, and *quality assurance.* In subsection 3, we will take a closer look at how the LMCs advance work in these areas, followed by a discussion of our findings in subsection 4. Below we will present our theoretical foundation and the methods used.

### Local medical centres in Norway

Norway has a long tradition of offering decentralised health care, for instance, through specialist doctors ambulating to local hospitals. This has been part of the larger national strategy of sustained regional development and equal access to health care across a geographically dispersed population. Increased decentralisation of health care has been a trend across the Nordic countries [13], and local community hospitals have been documented as an important element of the health care system since the 1980s [14, 15]. In Norway, the Coordination Reform has fuelled the focus on bringing specialist services closer to the patient and in a more flexible relationship to primary care services [16]. Patients now arrive from the hospital to the primary health service level at an earlier stage in their treatment and with more extensive care needs [17].

An increasing number of LMCs have been established as intermediate units, or “1 ½ level health services”, between primary and specialist care. LMCs serve as both an inpatient and outpatient ward for the regional hospital, while at the same time being collocated with primary health care services [12]. As intermediary units, these centres work in close collaboration with both specialised health care hospitals and municipal health care providers. LMCs are based on cooperation and co-sponsorship between two or more municipalities and the specialist care providers in the region. The Coordination Reform has led to an increased focus on these intermediate units, not least through significant state grants stimulating collaborative initiatives between municipalities. In 2010 there were 12 LMCs across the four administrative health regions [8]. The number today is most likely significantly higher, but as definitions are unclear, the total number is uncertain [18].

### Theoretical basis

We will base our exploration of the problem formulation on institutional theory, as well as on general safety theories.

In the Norwegian context, LMCs is an organisational form on the rise that needs to find its place in the health care system after the Coordination reform. This actualises institutional theory as a general analytical framework. Organisational institutionalism is a multifaceted field with a long history [19]. Seen from an ecological point of view, the survival of organisations depends on how social norms and expectations from surrounding actors are met [20]. This argument builds on the insights from classical contributions in the field of institutional theory [21], which involves adding a symbolic approach to pure rational, efficiency considerations in the study of organisations. The institutional environment, that is, conventions and norms in organisations’ surroundings, influence how organisations are designed and structured.

An institutional perspective on health care organisations may involve taking a bird’s eye view on LMCs and seeing them as “principal players” [20], which aims to craft their role and disposition and establish themselves as central actors in the landscape of health care organisations. Institutional perspectives explore why some types of organisations flourish, while others fade out and disappear over time. Scott et al. [20] presents *legitimacy* as a general explanatory mechanism for the ebb and flow of different forms of health organisations, and presents a classical institutional argument in this way: “Organizations require more than material resources and technical information if they are to survive and thrive in their social environments. They also need social acceptability and credibility” (ibid: p.237). Survival over time thus involves creating a legitimate role, which others trust. According to Zucker [22], institutions play an important role in the production of trust. Institutional trust has to do with how we trust societal institutions, such as hospitals, both in terms of professional roles and as specific organisations.

Following this argument, LMCs need to be perceived as legitimate parts of the health care system in order to prosper. In Norway, alignment of their activities with the Coordination reform is likely to contribute to their legitimacy, but LMCs must also be accepted and perceived as credible by specialist and primary health care.

**Fig. 1:**
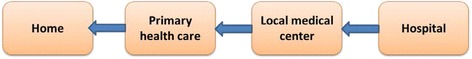
Shifting emphasis in the chain of care

A goal in the Coordination reform is to make a shift in chains of care “from right to left” in the treatment chain, as illustrated in Fig. 1. This implies that more of the treatment should be given locally and closer to the patient’s home environments. The coordination between the different domains in which health care is provided depends on the availability and quality of information, as well as comprehension of this information by the involved professionals. Thus, the *interfaces* between the actors in Fig. 1 are essential.

The Coordination reform involves a shift in the chain of care from hospitals to local environments.

In earlier research on safety in complex organisations, lack of information and breakdown in information flows is a common way to explain adverse events, as in the theory of “Man-made disasters” [23, 24]. In this perspective, accidents are not regarded as isolated events, but as *processes*, developing from a normal state to an “incubation period”, where information is lost, misunderstood, simplified and distorted, eventually leading to a precipitating event. The significance of information, both in terms of its availability and its quality, may thus be considered central to safety, also related to patients in treatment. The relevance of an information perspective on patient safety is demonstrated in numerous studies (see [25] for a review). For example, Dahl et al. [12] found that discharge of elderly patients from hospital via intermediary care units was favoured over the direct transfer from hospital to primary health care. These units represent additional resources for preparing patients for primary care, but professionals called for better information from the preceding care level [ibid.].

The competence of health care providers is also frequently studied in relation to patient safety [26–28]. In Norway, building more specialised competence in the primary care services is central as the Coordination reform has resulted in patients being discharged earlier from hospitals. They now arrive in the primary health service with more complex clinical needs. It also seems that the demand for increased knowledge has put a strain on professionals’ existent training, and medical equipment new to the primary care service creates a need for more training of personnel [29].

Patient safety is often related to the quality of health care. The Norwegian internal control regulations for Health, Safety and Environment (HSE) [30] requires systematic efforts to ensure HSE, including clear lines of responsibilities, mapping, and monitoring of hazards and risks and the compositions of measures to mitigate risk. The regulations are clearly inspired from general quality theory [31] and the idea of continuous improvement by means of the cyclic process of Plan-Do-Check-Act [32] is also made evident in international ISO standards. In health care, quality assurance systems may include external or internal audits, patient surveys, and reporting of adverse events.

Both information and competence and quality assurance will be applied as analytical categories in the analysis of the role of LMCs in subsection 3 (Results).

## Methods

The empirical material stems from a project evaluating the Coordination reform in Norway financed by the Norwegian Research Council (grant number 229623). We have concentrated our data gathering on one of the four health administrative regions in Norway. The main informants for this article are managers at three LMCs, managers at three municipal nursing home institutions, and the leader of a network established for managers at LMCs in this particular health administrative region. In addition to these seven main informants, thirteen other interviews were conducted, involving information and communications technology (ICT) coordination experts, patient association representatives, representatives from municipal authorities, and the patient ombudsman in this region. These interviews have provided a basis for understanding the complex coordination of health services, the regional context, and the importance of the LMC’s for other health care actors. The interviews were completed in accordance with the prevailing ethical standards, and the project is approved by The Data Protection Official for Research in Norway . The participants were contacted by e-mail and telephone. All participants received written and oral information about the study, including ethical standards for preserving anonymity and the right to withdraw from the study at any time. A confirmation was received from the participants by e-mail. Information about ethical procedures was repeated at the beginning of each interview.

The qualitative interviews were semi-structured and conducted in the period from May 2014 to January 2015. An interview guide with open-ended questions was used to be able to reveal unexpected perspectives [33]. The interview guide consisted of themes that provided knowledge about coordination of health care in the region and patient safety. Ten of the twenty respondents were interviewed face-to-face at their work places, and the others were interviewed by telephone or videoconference. All interviews were audio-recorded and transcribed verbatim and anonymized.

All authors independently read the transcriptions of the interviews to get an overview of the material and to identify themes related to the position of LMCs in the landscape of health care providers. Together, all authors discussed the material and developed categories encompassing how LMCs craft their intermediate position between hospitals and municipal care services in the promoting of patient safety practises and systems.

## Results

During the analysis, we constructed three categories that dealt with the role LMCs play in relation to patient safety. The categories were (1) information exchange, (2) competence development, and (3) quality assurance. Results related to the categories will be presented in the following.

### Information exchange: workarounds for accessing patient information

We find that the LMC has a role in the exchange of information between the primary and specialist care levels through their access to patient records in various ICT systems. Much of this access has been granted as workarounds but is considered vital for patient safety.

The municipalities that share the ownership of the LMCs have separate ICT systems and no access to each other’s patient records; neither do they have access to the hospitals’ system. The LMCs thus need to report and document in several different electronic patient record systems, one for each specific care provider at the municipal level.

One of the LMCs included in our study has access to both the system of the regional hospital and the different municipal systems. This LMC has, in cooperation with the hospital administration, found ways to access the hospital patient record system through workarounds. This is done by granting nurses at the LMC dummy positions (0 % positions) or registering them as temps at the hospital. This practise allows the nurses to access information in patient records without the delay involved in transferring the information from the specialist to the municipal system, and also to document what has been done at the centre. LMC managers emphasise the importance of this practise for their ability to provide health care, but they also refer to clinic managers at the hospital who indicate that collaboration with the LMC is easier when they work in the same patient record system.

However, this practise with dummy positions is at the discretion of the hospital administrators, and the interviews revealed different attitudes towards this practise across hospitals. One hospital did not allow these workarounds. This was partly explained as related to the fact that the LMC nurses are employed at the municipal level and, therefore, are not considered the responsibility of the hospital. LMC managers point out the drawbacks of this position, as the patients arriving at the LMC are stable but still in need of hospital care, and that the hospital patient record system contains crucial information for the nurses at the LMC. In the words of one LMC manager:

“It simply represents a hazard to the patient that we do not have a shared documentation system” (LMC manager 2).

Also, in the cases in which the hospital does not grant access for LMC nurses, there are other workarounds in use to provide a safe and efficient service. For instance, in the cases where the LMC offers dialysis treatment as a decentralised hospital service, this must be documented in the hospital system, and a special computer has been allocated for this purpose.

### Information exchange: local presence and local knowledge

An aspect of information exchange, which is more rarely addressed in terms of patient safety, is the relevance of information outside of what is documented in the patient record. We find that the employees at the LMC have access to information that may strengthen patient safety through knowledge of local conditions and available resources. Also, established interpersonal relations and trust between health care personnel allows the LMC flexibility to answer immediate needs.

In interviews with LMC and municipal personnel, the role of local knowledge is emphasised, both concerning the patients themselves and the resources available locally. Vulnerable elderly or cognitively weak patients are mentioned as patient groups that benefit from staying in the LMC rather than being transported to a larger hospital ward. Both the distance from familiar surroundings and the distance from those who know them, as well as the larger size of the hospital wards, are highlighted as relevant for the quality of care offered and for the patient’s sense of safety. With close collaboration between LMC and municipal care, these patients can receive services locally rather than having to travel to the regional hospital.

Through their collaboration with municipal services, frequently situated close to local/regional services and health administration offices, the LMCs have access to general patient information relevant for the assessment of needs. This can be information related to the patient’s home situation, such as whether the home is adequately equipped for returning home, whether s/he has responsibilities for the care of other family members, etcetera. Access to this information allows the LMC, in collaboration with the health administration office, to define appropriate and suitable services for the individual patient based on a description of the patient functionality and life situation as a whole, not only based on diagnostic information.

One LMC manager describes the work at the LMC as more focused on the patient’s ability to move and function than is the case in a hospital unit. With a physical therapist working directly with the patients on the bed post, more patients can be sent directly home from the LMC, thus reducing the need for municipal care.

The close communication between LMC and municipal services might in some cases also reduce the need for admittance to a nursing home, for example, in the case where the LMC orders a wheel chair for the patient so that s/he can go directly home after the stay in LMC, instead of sending the patient to a municipal nursing home while they are waiting for the wheel chair or other resources that can facilitate the return to home. This is very much in line with the objectives of the Coordination Reform, and it is facilitated by the close contact that the LMC creates between primary and specialist services.

A nursing home manager describes the transition from LMC to the nursing home in terms of a short phone call from the LMC directly to the nursing home, informing them that “Ola is coming home now”. ‘Ola’ was a patient already living at the nursing home before he went to the hospital. The familiarity, which the LMC has to both patients and local health care personnel, is described as contributing to both quality of care as well as to quality of information.

Since the LMC staff knows the local health care services, they also know what the municipality can offer and what to expect in terms of services. For example, one municipality has reduced the use of nursing homes and offers instead various forms of assisted living. The employees at the LMC know what this entails for the services that are offered, whereas a regional hospital might assume the existence of nursing homes and define care needs based on lacking knowledge of the services available. The nursing home personnel describe that one of the benefits related to the establishment of the LMC has led to a reduction in the number of “Friday patients”, known as patients who arrive at the nursing home on Fridays, due to the hospital’s attempt to reduce the number of patients over the weekend. “Friday patients” often arrive without clear descriptions or decisions regarding care needs.

The LMCs also benefit from the flexibility of local collaboration in realising safe patient care. As described by our informants, the LMC may to a certain extent juggle available resources to meet the immediate needs. This is made possible because of interpersonal relations and trust between health care personnel at the local level. This may, for instance, involve borrowing a bed belonging to the hospital when short term municipal beds are not available.

With a closer dialogue between service levels, the situation and needs of the patient are clarified, and the care transitions are consequently better coordinated. The patients arriving to municipal care are expected and known, including their needs in the municipal units in terms of personnel, competence, equipment, and resources. One municipal care manager simply states about the LMC that, despite initial scepticism to an additional service level, “we couldn’t manage without it”.

### Competence and training

While information flow and system support is of crucial importance for patient safety, the competence of health care staff also plays a significant role. The Coordination reform, with its emphasis on early discharge from hospital and more advanced treatment at the municipal level, represents a challenge for the municipalities in terms of competence.

The LMCs are seen as a resource for strengthening the competence and training of municipal health personnel. Nurses from municipal institutions are invited to have internships at the LMC, which gives them the opportunity to learn about complex patient cases and work procedures. The LMC also sends out specialist personnel to the municipalities, for audits and/or specific training. For example, the hygiene nurse was visiting nursing homes in the municipalities, auditing their routines on hygiene, while also offering on-site training and information. A local diabetes nurse at the LMC offered training to healthcare personnel at the nursing homes in the area (see also [34]). The LMCs are also hosting weekly and monthly lectures and training that are open for the municipal units to attend via videoconference. In addition, they often manage health projects that include hospital units, municipal services, resource centres, patients, and families that aim to increase knowledge and learning related to specific conditions, such as chronic obstructive pulmonary disease or cancer. Health promotion groups offer training and support on topics such as physical activity, diet, smoking, and living with illness. One of the LMC managers also offered her own training programmes on specific illnesses and medication management.

In municipal health services, as much as 29% of the labour is performed by personnel without appropriate formal health professional education, mostly in long-term care [17]. In this situation, we find that LMCs provide and develop needed competence on the local level.

### Quality assurance

We find that the LMCs quality and patient safety work take advantage of the resources that are already available in the hospitals, as well as create new patient safety measure.

The LMCs work in the municipal Quality assurance (QA) system, following general existing routines for the reporting of unwanted events. However, the LMC managers and most of the other personnel have experience from specialised care services and the hospital domain, bringing with them procedures and practices from specialist care into the LMC. One manager describes her involvement in launching the LMC and how she established new routines and procedures for patient safety, inspired by her personal experience in hospital work, such as checklists, routines for learning, follow-up, and compliance. The managers in our interviews describe a great number of local initiatives being taken by the LMC, in terms of creating new routines and procedures. One LMC manager has created a local quality team that resembles the standard quality committee in specialist care, as a way of filling the gap she experiences in patient safety work. Another LMC has employed a pharmacist once a week for checking medicine lists.

No national or standardised routines have been available on the municipal level, although following the current national program for patient safety, such resources are emerging and gradually becoming more accessible.

The municipal health care does follow municipal regulations for quality control of services, and routines are in place for ensuring that standards are met and that unwanted events are reported. However, the reports filed in this system are not standardised and do not reach a national or regional level, in which this information can be aggregated and used for learning and improvement. There is no addressee for these reports of adverse events, except for the local-institutional and municipal management. Therefore, there is little general knowledge of the extent or content of local adverse events.

## Discussion

### LMCs, legitimacy, and patient safety

**Fig. 2:**
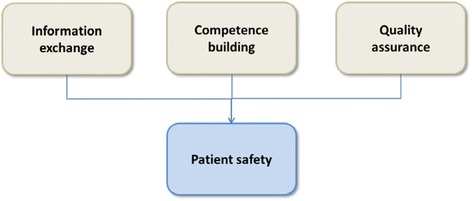
Local medical centres’ role in improving patient safety

Seen in light of institutional theory, LMCs have to carve out and legitimise their role in the health care system and align it with the Coordination reform. Our analysis indicates that LMCs gain legitimacy by (1) mediating information between health care levels, and (2) by providing competence, and (3) by supporting quality assurance at the local level. All these three aspects have the potential to strengthen patient safety (Fig. 2).

Local medical centres are found to contribute to patient safety through improving information exchange, competence building and quality assurance.

Related to the Coordination reform, several new LMCs have been established. One might argue that this has increased the complexity of the health care system, involving more organisational interfaces and more opportunities for things to go wrong, information to get lost or distorted, etcetera. In organisational safety theory, complexity is seen as a condition that increases the probability of adverse events, as in Perrow’s theory of normal accidents [35]. Still, our findings indicate the opposite, that LMCs increase the robustness of the health care system. Lending the concept of *organisational redundancy* from the theory of high reliability organisations [24, 36], overlapping competencies, tasks, and responsibilities between different levels of health care might increase the probability of errors to be intercepted. LMCs can in this way be seen to serve as an extra barrier against patient hazards.

Rosness et al. [24] highlight a *cultural* dimension of organisational redundancy, involving “…the capability and willingness to exchange information, provide feedback, reconsider decisions made by oneself and colleagues, and intervene to recover erroneous actions” (ibid: p.58). LMCs can be seen as carriers of such cultural dimensions. They have a role to play in the gap between primary and specialist culture and rationality. They represent a unit in the interface between the two health care levels, where professionals from both levels meet. Krasnik and Paulsen [13] point out that reform work in both hospitals and the municipal health services has been triggered in each sector by its own culture and organisational rationality, leaving many important questions of coordination between them unresolved. As LMCs work across care levels, they can function as an accelerator in the development of a robust patient safety culture in the municipalities through their close link to the practises and routines of specialist care-both because of exchange of personnel and the mutual competence building between municipal and LMC nurses.

A sign of the legitimacy of LMCs is that they, according to those interviewed, have become popular workplaces for competent health personnel who might otherwise have worked at the regional hospital. At the LMC they get similar work challenges as in the hospital and also an opportunity to work closer to home. With the structured activities for competence building that includes the municipal health care personnel, the LMC creates arenas and opportunities for continuous learning beyond the single institution or unit.

### Connecting levels of care

Several factors make it challenging to create collaborative chains across levels of health care [37], including different ICT systems and incongruent procedures, but also power difference and asymmetries between specialist and primary care services. Also, different organisational cultures between the two service levels make it challenging to create effective collaborative chains [38].

We find that LMCs might function as a bridge between levels of care, showing significant insights into both specialised and primary services and finding practical solutions for easing the transition between the levels. Seen in light of an information perspective [23, 24], this might be important for strengthening patient safety. A critique that has been put forward is that patient safety initiatives have been too hospital-centred, and too process-and ‘silo’-driven [39]. The LMCs bridge-building function might contribute to a needed reinforcement of the involvement of the primary care level in patient safety work.

The importance of LMCs and their bridge-building function should also be seen in light of the fact that there are currently not adequate systems and tools to support integrated care pathways and the sharing of information across organisations and professionals in the Norwegian health care sector. Work processes and care plans are not standardised; consequently, a great deal of coordination across service levels and units is done by telephone or paper documents sent by post. Paulsen et al. [37] found that hospital information provided at discharge was neither sufficient nor timely. Deficits in the information exchange between service levels will have a negative impact on patient safety when patient-related information is lost, distorted, or misunderstood. The LMCs access to the different ICT systems might mitigate the risk for harm and contribute to more correct and updated information following the patients.

### Limitations and future research

The empirical basis is limited to one administrative health region and involves three LMCs and nursing homes, in addition to informants from one administrative office. Thus, the study is not representative for all regions and LMCs in Norway. Also, current LMCs are highly diverse, and the present study may not capture this heterogeneity. Nevertheless, the study represents a case that illustrates how the LMCs can strengthen patient safety practises and competence in the interface between primary and specialist health care. This role of LMCs might be of general value, as efforts to integrate health care systems are evident in many countries [6].

There is clearly a need for more systematic knowledge about the structure and functions of the LMCs. Further research should explore the role of LMCs in patient safety work across other administrative regions and countries. The views from representatives from specialised care professionals will also be a valuable supplement when exploring the role of LMCs in patient safety. It may also be fruitful to include general practitioners, physical therapists, and other health professionals working in the municipal health services.

## Conclusions

In terms of patient safety, the specialist health care level has worked extensively with patient safety the last decade, through national as well as regional campaigns. The primary care level, on the other hand, has worked less systematically with this topic and is currently not represented in national statistics or reporting systems in Norway. The current patient safety program (2014–2018) has ambitions to bring patient safety work more to primary health care in the same structured ways as has been the case in specialist health care. The national goal is set for 25 % of all municipalities working actively with patient safety within 2018. The current study sees the Local Medical Centres as a potential resource in this effort.

Local Medical Centres were originally developed in regions in which the population was spread far away from the central hospital. With the recent Coordination reform, these centres have gained increasing relevance and are seen as important sites for the integration and coordination between primary and specialist health care services.

Fragmentation of care, increased number of interfaces, and care transitions could be seen as posing a threat to patient safety. The current study approaches this additional level of health care, in between primary and specialist, rather as a potential resource in the continuous work on improving quality of care and patient safety.

With their position in the interface between primary and specialist care, LMCs can serve a role as quality assurers, local competence providers, and bridge builders in terms of patient safety attitudes and practises. With increased awareness of this role, the LMCs might strengthen their legitimacy as an actor in health care. Primary care services might use the experience and practises in the LMCs to further their efforts to strengthen patient safety systems.

More systematic information about the LMCs is needed, regarding personnel, competence, and responsibilities, in order to thoroughly assess and optimise their role in the interface between specialist and primary care. Based on the current study, we believe that the LMSs can play an important role in bridging the gap between care levels and strengthen patient safety in integrated care pathways. This might be applicable for other regions and countries, although we need more research in other contexts.

## Abbreviations

EU: European union
HSE: Health, safety and environment
ICT: Information and communications technology
ISO: International organization for standardization
LMC: Local medical centre
QA: Quality assurance
WHO: World health organization
